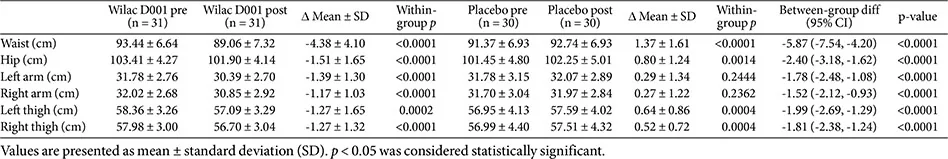# Effects of Wilac D001 (*Weissella confusa* WIKIM51) Supplementation on Metabolic Parameters and Central Adiposity in Overweight Adults: A 12-Week Randomized, Double-Blind, Placebo-Controlled Trial

**DOI:** 10.4014/jmb.2606.06054

**Published:** 2026-09-02

**Authors:** Sangmin Park, Byungwook Lee, Hwayeon Sun, Wangyoon Shin, Ji-ye Mok, Jeonghyun Seo, Hak-jong Choi, Ga Hee Choi, Ju Yeon Son, Byungwook Yoo

**Affiliations:** 1 Microbiome R&D Center, Pharmsville Co., Ltd., Seoul 07793, Republic of Korea; 2 Kimchi Healthcare Research Group, World Institute of Kimchi, Gwangju 61755, Republic of Korea; 3 Division of Medical Science, Soonchunhyang University, Asan, Republic of Korea; 4 Department of Family Medicine, Soonchunhyang University Seoul Hospital, Seoul 04401, Republic of Korea; 5 Department of Pharmacy, Seoul National University, Seoul, Republic of Korea

**Keywords:** Probiotics, Metabolic health, Glucagon-like peptide-1, *Weissella confusa*

## Abstract

Recent research has emphasized the importance of gut microbiota in regulating energy and lipid metabolism as well as gut hormone secretion, indicating that probiotics could contribute to improving metabolic parameters associated with excess adiposity. This study evaluated the metabolic effects of *Weissella confusa* WIKIM51 (Wilac D001) and explored the potential involvement of glucagon-like peptide-1 (GLP-1)–related mechanisms. First, GLP-1 secretory activity following Wilac D001 treatment was evaluated *in vitro* using STC-1 enteroendocrine cells. Male C57BL/6 mice were fed a high-fat diet to induce obesity-associated metabolic dysregulation, and Wilac D001 was orally administered for 10 weeks. Glucose tolerance was subsequently examined using an oral glucose tolerance test (OGTT). Finally, in a 12-week randomized, double-blind, placebo-controlled clinical trial, overweight adults aged 19–64 years received either Wilac D001 (1.0 × 10^10^ colony-forming units/day) or a placebo. Blood lipid parameters, glycated hemoglobin, abdominal fat distribution, body composition, body weight, regional anthropometric measures, and lifestyle factors were evaluated. Wilac D001 significantly increased GLP-1 secretion in STC-1 cells versus the control (*p* < 0.01). High-fat diet-fed mice administered Wilac D001 exhibited lower fasting blood glucose levels and more rapid glucose recovery during the OGTT (*p* < 0.01). In the clinical trial, Wilac D001 supplementation led to reductions in several markers, including a significant reduction in triglyceride levels, alongside improvements in total abdominal fat area, visceral fat area, and subcutaneous fat area. No serious adverse events were reported. These findings support the potential of Wilac D001 as a functional probiotic for promoting microbiome-based metabolic health management.

## Introduction

Metabolic disorders, including obesity, type 2 diabetes mellitus, dyslipidemia, and nonalcoholic fatty liver disease, are major chronic diseases with increasing global prevalence and are recognized as major risk factors for cardiovascular disease and various related complications [1, 2]. Moreover, these metabolic disorders are closely associated with complex metabolic imbalances, including impaired glucose and lipid metabolism, chronic low-grade inflammation, and excessive fat accumulation. In recent years, the prevalence of metabolic disorders has increased rapidly owing to Westernized dietary patterns, reduced physical activity, and population aging, leading to growing interest in their prevention and management [3]. Recent studies have reported that gut microbiota dysbiosis can induce metabolic inflammation and play an important role in the development and progression of adiposity and metabolic disorders [4, 21]. Specifically, excessive nutrient intake and certain dietary patterns can alter the composition of the gut microbiota, and these alterations may be associated with various metabolic abnormalities, including dysregulated lipid metabolism and inflammatory responses [5]. Such changes in the gut microbiota have also been closely linked to obesity, type 2 diabetes mellitus, and cardiovascular disease [6].

*Weissella confusa* WIKIM51 (Wilac D001) is a lactic acid bacterium isolated from dandelion kimchi produced in Namhae, Korea. Previous studies have demonstrated that Wilac D001 suppresses the expression of lipogenesis-related genes, including sterol regulatory element-binding protein-1 and fatty acid synthase, and reduces lipid accumulation *in vitro* [7]. Additionally, *in vivo* studies using a rat offspring model of metabolic impairment induced by maternal high-fat diet exposure and postnatal dietary intervention reported that Wilac D001 administration reduced hepatic triglyceride accumulation, alleviated oxidative stress, and suppressed hepatic lipogenesis [8].

Furthermore, increasing attention has recently been focused on the role of gut microbiota-derived metabolites and the gut–metabolic axis in the regulation of appetite and metabolism [22]. In this context, the gut hormone glucagon-like peptide-1 (GLP-1) has emerged as an important regulatory factor. GLP-1 regulates glucose homeostasis and lipid metabolism by stimulating insulin secretion, delaying gastric emptying, and suppressing appetite. Several studies have suggested that specific probiotics or their derived components can enhance GLP-1 secretion in enteroendocrine cell models and that such changes may be associated with improvements in body weight and metabolic parameters [9-11].

In addition to the preclinical evidence described above, the clinical efficacy of Wilac D001 has previously been investigated in overweight and obese adults [12]. However, further studies are warranted to provide a more comprehensive evaluation of its effects on obesity- and metabolism-related outcomes. In particular, the effects of Wilac D001 on regional adiposity-related anthropometric measures and glycemic control markers, such as glycated hemoglobin (HbA1c), have not been sufficiently characterized. Moreover, the assessment of regional anthropometric measures, including upper-arm circumference and thigh circumference, in addition to abdominal fat distribution, may provide a more comprehensive understanding of the potential effects of Wilac D001 on body composition and metabolic health.

## Materials and Methods

### *In Vitro* GLP-1 Secretion Assay

**STC-1 cell line culture.** STC-1 is a mouse-derived intestinal enteroendocrine cell line that exhibits characteristics of L-cells, which secrete incretin hormones such as GLP-1. In this study, STC-1 cells were used as an experimental model to evaluate the ability to induce GLP-1 secretion. STC-1 cells were cultured in Dulbecco’s Modified Eagle Medium supplemented with 10% fetal bovine serum, 100 U/mL penicillin, and 100 μg/mL streptomycin. Cells were maintained in a humidified incubator at 37°C under 5% CO_2_, and the culture medium was replaced every 3 days. When cell confluence approximately reached 70%–80%, cells were detached using 0.25% trypsin-EDTA (ThermoFisher Scientific, USA) and collected by centrifugation. The cells were then evenly seeded into six-well plates at a density of 2 × 10^6^ cells per well. Experiments were conducted when cell confluence reached approximately 80% 2 days after seeding.

**Probiotic culture and viable count test.** Wilac D001 was activated on *Lactobacilli* MRS agar plates, and a single colony was selected and cultured in liquid medium. For viable cell count analysis, the optical density of the liquid culture at 600 nm was adjusted to 1.0. Furthermore, the culture suspension was serially diluted using a 10-fold dilution method, and each dilution was plated and incubated for 24 h. Plates containing 20–200 colonies were selected, and colony-forming units (CFU) were calculated accordingly. Live bacterial cells were washed with phosphate-buffered saline (PBS) and subsequently used in the assay.

**Assessment of GLP-1 secretion post-probiotic treatment.** To evaluate GLP-1 secretion, STC-1 cells were used at approximately 80% confluence. Cells were washed twice with a buffer solution lacking additives, such as glucose or L-cysteine, and then incubated for 3 min to induce a transient starvation state. Subsequently, cells were incubated for 15 min with phosphate-buffered saline (negative control), PBS-washed live Wilac D001, *Escherichia coli*, lipopolysaccharide (LPS), or docosahexaenoic acid (DHA; positive control) at a concentration of 2 × 10^7^ CFU per well. After incubation, the culture medium was collected into tubes containing a DPP-IV inhibitor and centrifuged at 800 ×*g* for 5 min. The supernatant was then harvested, and GLP-1 concentrations were measured *via* enzyme-linked immunosorbent assay (ELISA) (Multispecies GLP-1 ELISA Kit, Cat. No. BMS2194; Thermo Fisher Scientific). Although STC-1 cell viability was not quantitatively assessed, no apparent cell death was observed by microscopic examination during the 15-min exposure to Wilac D001.

### *In Vivo* Oral Glucose Tolerance Test

**Animals and experimental design.** Five-week-old male C57BL/6 mice were purchased from Orient Bio (Republic of Korea) and acclimated to the housing environment for 7 days before the start of the experiment. The animals were then assigned to a normal diet group (n = 8), a high-fat diet (HFD) group (n = 8), or an HFD plus Wilac D001 group (n = 8). Wilac D001 was orally administered to the HFD plus Wilac D001 group at a dose of 1 × 10^9^ CFU/200 μL, five times per week for 10 weeks. An oral glucose tolerance test (OGTT) was performed during week 8 of probiotic administration. All experimental procedures were approved by the Institutional Animal Care and Use Committee of the World Institute of Kimchi (Approval No. WIKIM-IACUC-202606).

**Animal housing conditions.** The mice were housed under controlled environmental conditions (temperature, 22°C ± 2°C; relative humidity, 50% ± 10%; 12-h light/12-h dark cycle), with 4–5 animals per polypropylene cage. The normal diet group was fed a standard rodent chow diet, whereas the HFD groups were fed a high-fat diet containing 45% of total energy from fat *ad libitum*. Cages and sterilized drinking water were replaced weekly.

**OGTT.** Blood glucose levels were measured during week 8 of probiotic administration. Before testing, the mice were fasted for 18 h and then orally administered glucose at a dose of 2 g/kg body weight. Blood glucose concentrations were subsequently measured from tail vein blood samples at 0, 20, 60, 90, and 120 min after glucose administration to assess glucose tolerance.

### Clinical Trial Study Design and Participants

The 12-week randomized, double-blind, placebo-controlled clinical trial was designed as a parallel-group trial with participants randomly assigned in a 1:1 ratio to either the Wilac D001 supplementation or placebo group. This study was conducted from April 2024 to March 2025. The study was conducted in accordance with the Declaration of Helsinki (2013) and the International Council for Harmonisation Good Clinical Practice guidelines. The study protocol was approved by the Institutional Review Board of Soonchunhyang University Hospital (SCHUH IRB 2023-11-016) and prospectively registered with the Clinical Research Information Service (registration no. KCT0011174). Adults aged 19–64 years with a body mass index (BMI) of 25–30 kg/m^2^ were eligible. Detailed inclusion and exclusion criteria are provided in the Supplementary Appendix (Table A1).

**Randomization.** Participants who met the predefined inclusion and exclusion criteria were randomly assigned to either the intervention or placebo group using a block randomization method to ensure balanced allocation between groups. The randomization schedule was generated prior to study initiation using the SAS^®^ program (version 9.4; SAS Institute, USA) and allocation codes were assigned sequentially according to the randomization schedule and screening order. Randomization codes were maintained by an independent researcher, and they were not disclosed to the participants or investigators until completion of the study.

**Study procedures.** Participants in the intervention group consumed one capsule containing 1.0 × 10^10^ CFU of Wilac D001 once daily for 12 weeks, whereas participants in the placebo group consumed an identically appearing capsule containing maltodextrin once daily. All participants completed four hospital visits: screening (visit 1), randomization (visit 2), mid-intervention visit at week 6 (visit 3), and end-of-intervention visit at week 12 (visit 4). At each visit, participants returned any remaining capsules and received a new supply of the assigned treatment. Adverse events occurring during the study period were communicated verbally and continuously documented throughout the trial. Compliance was evaluated according to the number of returned capsules and participant self-reports, and compliance was considered acceptable when participants consumed at least 80% of the assigned capsules.

Blood samples were collected under fasting conditions to analyze lipid parameters (total cholesterol, HDL cholesterol, LDL cholesterol, and triglycerides), glycated hemoglobin (HbA1c), aspartate aminotransferase (AST), and alanine aminotransferase (ALT). Body composition analysis was performed using dual-energy X-ray absorptiometry (DEXA) to measure total body fat mass, body fat percentage, and lean body mass. Abdominal fat distribution, including total abdominal fat area ratio, visceral fat area, subcutaneous fat area, and visceral-to-subcutaneous fat area, was assessed using computed tomography (CT). Anthropometric measurements, including height, body weight, BMI, waist circumference, hip circumference, upper-arm circumference, thigh circumference, and vital signs (systolic blood pressure, diastolic blood pressure, and pulse rate), were obtained at each study visit, including baseline.

Dietary intake and physical activity were assessed using 3-day dietary records and the Global Physical Activity Questionnaire, respectively. All participants received standardized lifestyle guidance recommending a daily reduction of approximately 500 kcal in energy intake and an increase of approximately 300 kcal/day in physical activity throughout the study. Changes in dietary intake and physical activity were assessed to evaluate adherence to the lifestyle guidance. For women of childbearing potential, pregnancy status was confirmed at the screening visit.

**Outcomes.** The prespecified primary outcome was the change in total body fat mass from baseline (Visit 2, Week 0) to the end of the 12-week intervention (Visit 4, Week 12), as assessed using dual-energy X-ray absorptiometry (DEXA; Horizon W, Hologic Inc., USA). Prespecified secondary outcomes included changes from baseline to Week 12 in body fat percentage and lean body mass; body weight and body mass index (BMI); waist circumference, hip circumference, waist-to-hip ratio, upper-arm circumference, and thigh circumference; total abdominal fat area, visceral fat area, subcutaneous fat area, and the visceral-to-subcutaneous fat area ratio; serum lipid parameters (total cholesterol, HDL cholesterol, LDL cholesterol, and triglycerides); glycated hemoglobin (HbA1c); and aspartate aminotransferase (AST) and alanine aminotransferase (ALT). Safety was assessed based on adverse events, clinical laboratory test results, vital signs, and physical examination findings.

**Safety assessment.** Safety assessments were conducted through continuous monitoring of adverse events throughout the study period. Safety endpoints included vital signs (blood pressure and heart rate), laboratory test results, and self-reported symptoms from participants.

### Statistical Analysis

The sample size was calculated based on the prespecified primary outcome, the change in total body fat mass using data from Cho *et al*. [13]. Based on a pooled SD of 2.03 kg and a conservative between-group difference of 1.48 kg, with a two-sided α of 0.05, 80% power, and a 1:1 allocation ratio, 30 participants per group were required. Allowing for a 25% dropout rate, the target sample size was set at 40 participants per group (80 participants in total).

The primary efficacy analysis was performed in the per-protocol population, as prespecified in the study protocol. The PP population comprised participants in the full analysis set who completed the study without major protocol deviations that could substantially affect efficacy evaluation, including major violations of the inclusion/exclusion criteria or use of prohibited concomitant medications.

Continuous variables are presented as mean ± standard deviation (SD). Within-group changes in primary and secondary efficacy outcomes were analyzed using paired *t*-tests. For between-group comparisons of changes, normality was assessed using the Shapiro–Wilk test. A two-sample *t*-test was used when the normality assumption was satisfied, and the Wilcoxon rank-sum test was used otherwise. As prespecified in the study protocol, ANCOVA could be performed using relevant baseline demographic or lifestyle characteristics as covariates when statistically significant or clinically relevant between-group differences were identified. Accordingly, alcohol consumption, which differed significantly between groups at baseline, was included as a covariate in the relevant ANCOVA analyses. All statistical analyses were performed using SAS^®^ version 9.4 (SAS Institute, USA). All tests were two-sided, with *p* < 0.05 considered statistically significant.

## Results

### GLP-1 Secretion in STC-1 Cells

Changes in GLP-1 secretion following Wilac D001 treatment were evaluated in STC-1 cells. The Wilac D001-treated group exhibited a 1.84-fold higher level of GLP-1 secretion than the control group (72.584 ± 0.387 ng/mL per mg protein vs. 40.842 ± 5.473 ng/mL per mg protein; *p* < 0.01). The DHA-treated group exhibited the highest level of GLP-1 secretion (99.157 ± 5.587 ng/mL per mg protein), corresponding to a 2.55-fold increase versus the control group (*p* < 0.0001). Conversely, no significant changes in GLP-1 secretion were observed in the *E. coli*- or LPS-treated groups compared with the control group (Fig. 5).

### OGTT

An OGTT was performed to evaluate changes in glucose tolerance. Administration of Wilac D001 to mice with high-fat diet (HFD)-induced obesity significantly improved glucose tolerance. The HFD control group exhibited elevated blood glucose levels throughout the observation period, whereas the Wilac D001-treated group displayed lower fasting blood glucose levels, attenuated postprandial glucose excursions, and more rapid glucose recovery. Notably, blood glucose levels at 90 min after glucose loading were significantly lower in the Wilac D001-treated group than in the HFD control group (*p* < 0.05). Consistent with these findings, analysis of the glucose area under the curve (AUC) demonstrated a significant reduction in the Wilac D001-treated group compared with the HFD control group (*p* < 0.01; Fig. 6).

### Clinical Trial Results

**Fig. 1:**
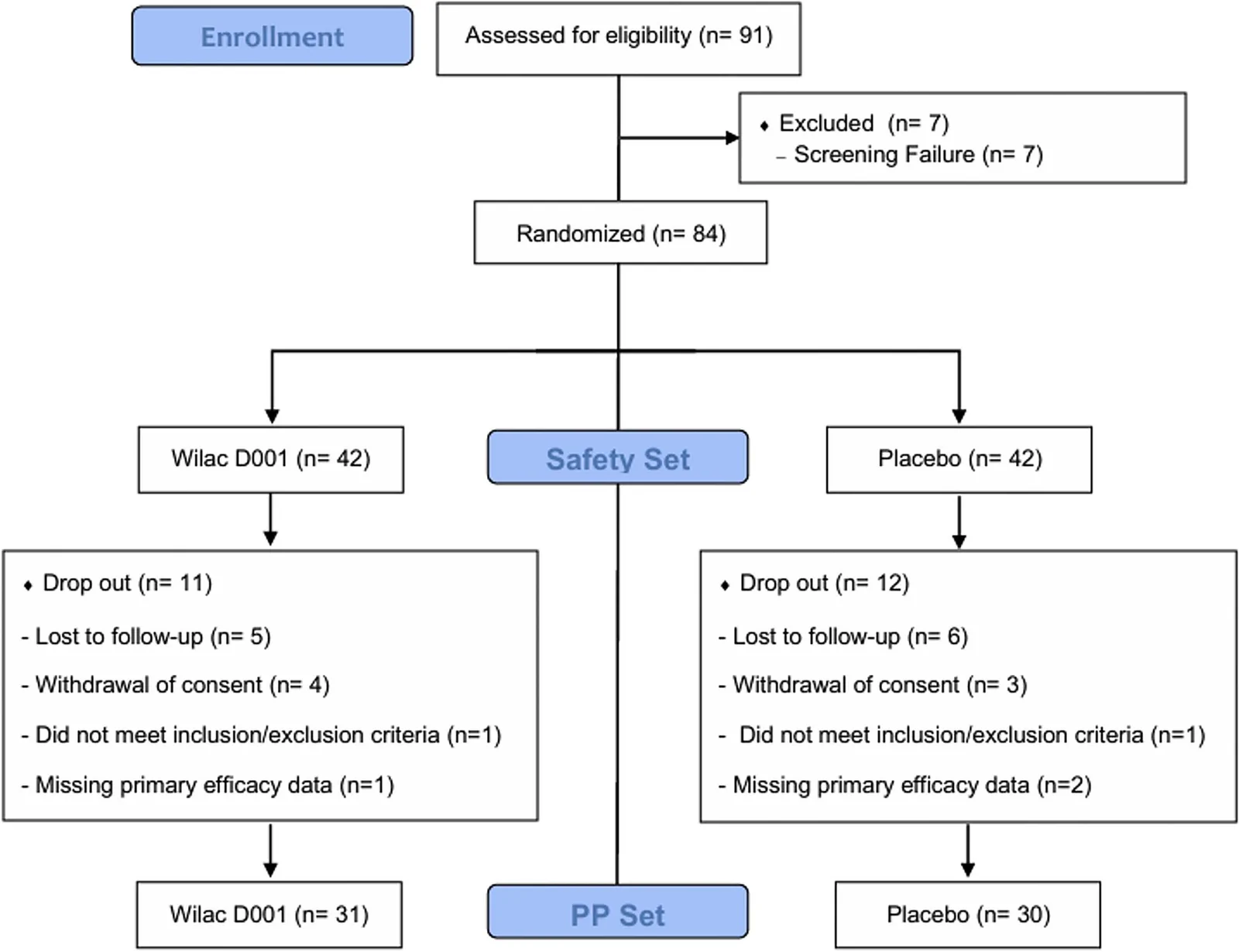
Flow chart showing the origin of the subjects included in this study, as well as the reasons for exclusion.

This study was conducted from April 2024 to March 2025. Overall, 91 participants were screened, of whom 84 were randomized. Ultimately, 61 participants were included in the per-protocol analysis (Wilac D001 group, n = 31; placebo group, n = 30). The numbers of participants excluded from the per-protocol analysis were comparable between the two groups (11 vs. 12), and withdrawal of consent was the most common reason for discontinuation (Fig. 1).

### Baseline Characteristics

The mean age was 37.29 ± 7.82 years in the Wilac D001 group and 37.07 ± 6.94 years in the placebo group. There were no significant differences in demographic or clinical characteristics between the two groups at baseline (Table 1).

### Changes in Body Weight and Body Composition

After 12 weeks of intervention, body weight decreased significantly in the Wilac D001 group (-3.38 ± 2.30 kg) but increased in the placebo group (+1.14 ± 1.59 kg), resulting in a significant between-group difference (adjusted mean difference: -4.40 kg; 95% CI: -5.45 to -3.35; *p* < 0.0001). A similar pattern was observed for BMI, which decreased in the Wilac D001 group (-1.18 ± 0.82 kg/m^2^) but increased in the placebo group (+0.37 ± 0.55 kg/m^2^), with a significant between-group difference (*p* < 0.0001).

Total body fat mass, the prespecified primary outcome measured by DEXA, decreased in the Wilac D001 group (-1,230.96 ± 1,851.38 g), whereas it increased in the placebo group (+450.75 ± 1,509.72 g). The adjusted between-group difference was -1,561.99 g (95% CI: -2,459.05 to -664.94; *p* = 0.0009).

Regional body fat mass also decreased significantly in the Wilac D001 group compared with the placebo group in the left arm (*p* = 0.0445), trunk (*p* = 0.0052), left leg (*p* = 0.0147), and right leg (*p* = 0.0107). No significant between-group difference was observed in the right arm (*p* = 0.1940) (Table 2).

### Changes in Metabolic Markers

**Fig. 2:**
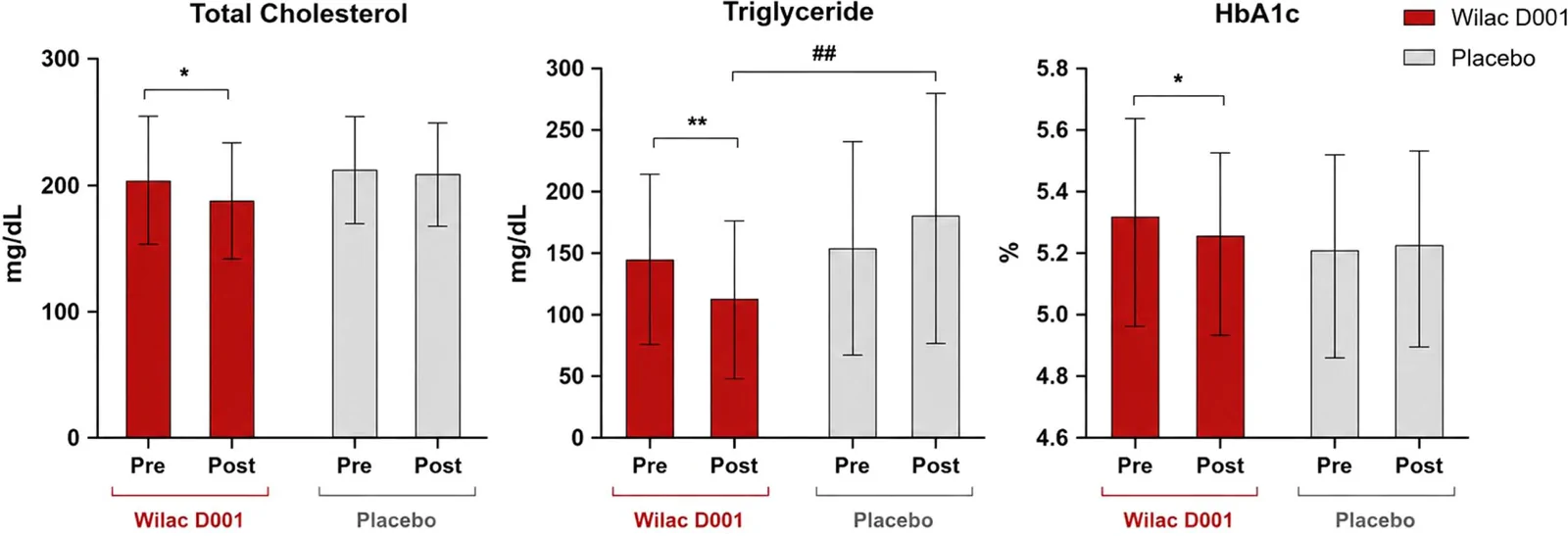
Changes in total cholesterol, triglyceride, and HbA1c levels after 12 weeks of supplementation. Values are presented as mean ± SD. Within-group comparisons were analyzed using paired t-tests, whereas unadjusted between-group differences were analyzed using two-sample t-tests or Wilcoxon rank-sum tests, as appropriate. *p < 0.05 and **p < 0.01 versus baseline within the same group; #*p* < 0.05 and ##*p* < 0.01 for unadjusted between-group differences.

Changes in blood lipid and glucose metabolism-related parameters are presented in Table 3 and Fig. 2. Triglyceride levels significantly decreased in the Wilac D001 group (-33.00 ± 61.28 mg/dL, *p* = 0.0054), with significant between-group differences in both the unadjusted (Wilcoxon rank-sum test, *p* = 0.0019) and ANCOVA-adjusted (*p* = 0.0018).

HbA1c significantly decreased from baseline in the Wilac D001 group (-0.07 ± 0.16%, *p* = 0.0133). The unadjusted between-group difference was significant (*p* = 0.0497), whereas the adjusted analysis showed a favorable, but non-significant, difference (*p* = 0.0632). Total cholesterol and LDL cholesterol also significantly decreased from baseline in the Wilac D001 group, although between-group differences were not significant.

ALT showed a significant unadjusted between-group difference (*p* = 0.0112), which was attenuated after ANCOVA adjustment (*p* = 0.2021). No significant between-group difference was observed for AST. Overall, the most consistent between-group improvement was observed for triglyceride levels, with favorable changes also observed in several other metabolic parameters (Table 3).

### Changes in the Abdominal Fat Area

**Fig. 3:**
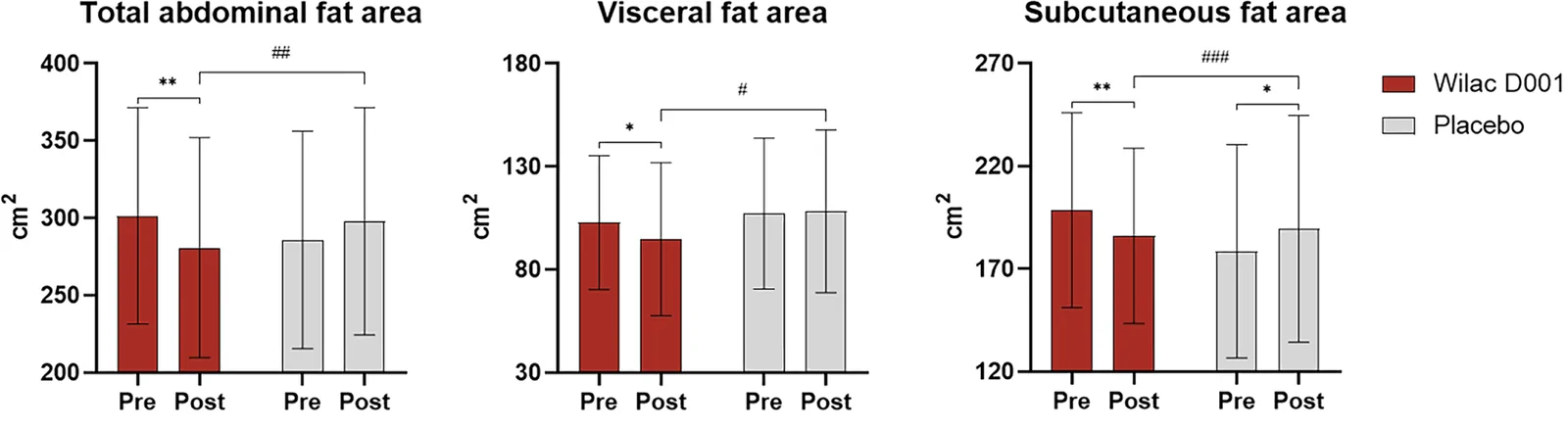
Changes in total, visceral, and subcutaneous abdominal fat area (cm^2^) assessed by CT after 12 weeks.

CT analysis demonstrated significant reductions in abdominal fat area in the Wilac D001 group compared with the placebo group after 12 weeks of supplementation (Table 4, Fig. 3). The total abdominal fat area significantly decreased by 20.57 ± 38.36 cm^2^ in the Wilac D001 group (*p* = 0.0056), whereas it increased nonsignificantly by 11.98 ± 32.37 cm^2^ in the placebo group (*p* = 0.0519), resulting in a significant between-group difference (*p* = 0.0012). Visceral fat area also significantly decreased by 8.09 ± 19.83 cm^2^ in the Wilac D001 group (*p* = 0.0305), whereas a nonsignificant increase of 1.10 ± 14.56 cm^2^ was observed in the placebo group (*p* = 0.6822), resulting in a significant between-group difference (*p* = 0.0493). Similarly, subcutaneous fat area significantly decreased by 12.48 ± 24.09 cm^2^ in the Wilac D001 group (*p* = 0.0072), whereas it significantly increased by 10.88 ± 24.57 cm^2^ in the placebo group (*p* = 0.0217), resulting in a significant between-group difference (*p* = 0.0008).

### Changes in Body Circumference

**Fig. 4:**
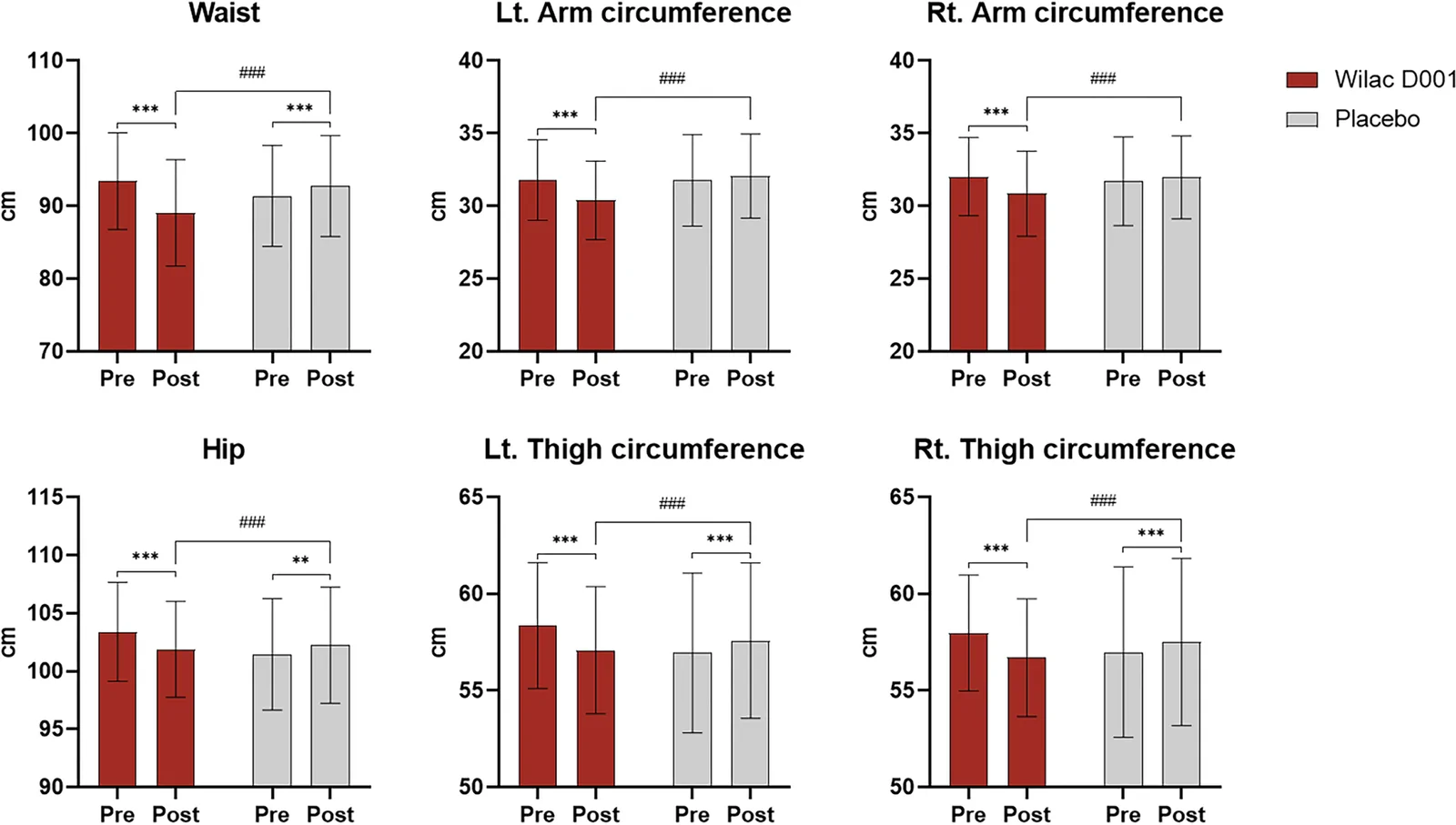
Changes in waist, hip, upper arm, and thigh circumferences.

**Fig. 5:**
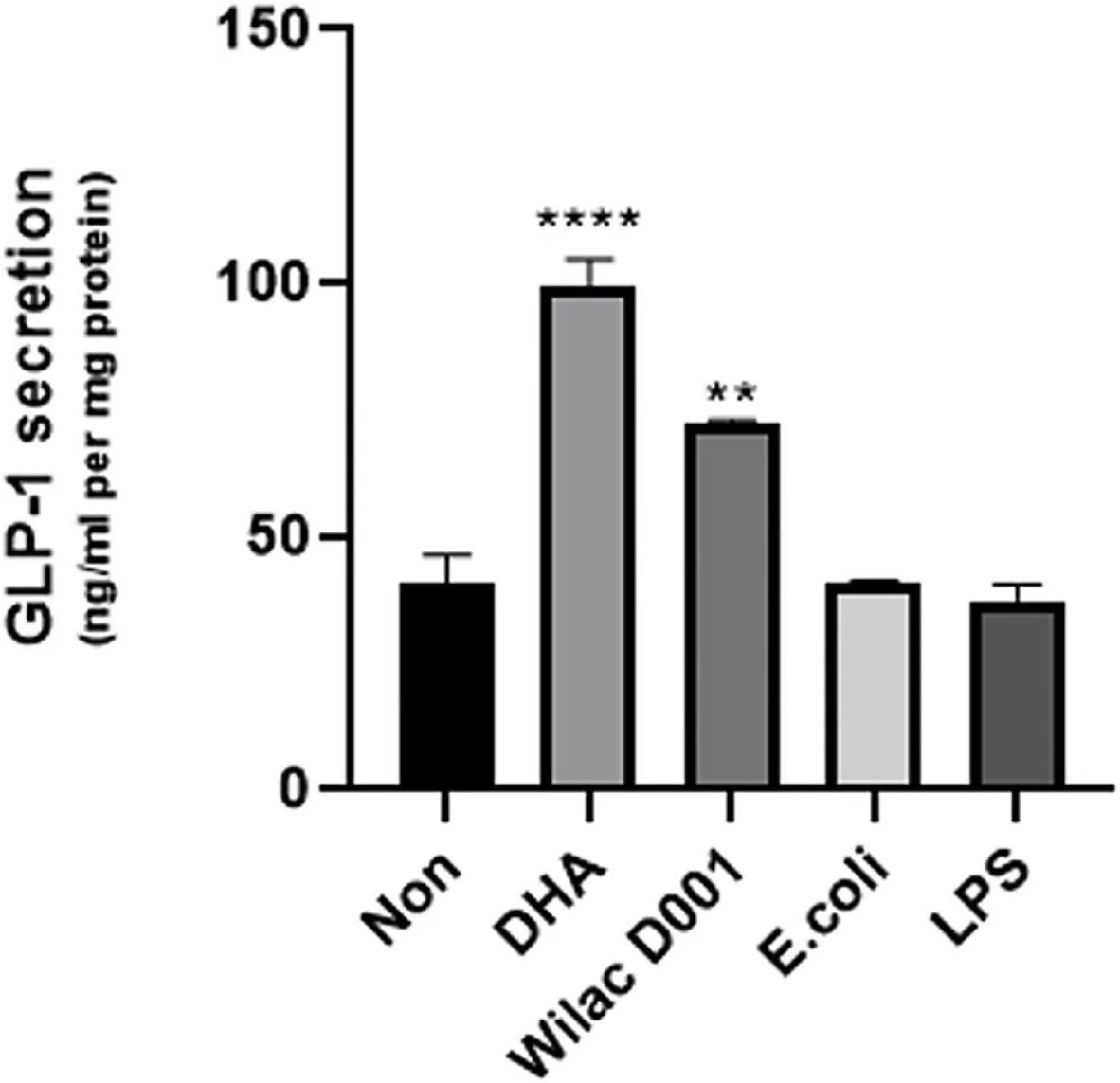
GLP-1 secretion levels in STC-1 cells after treatment with different stimuli. STC-1 cells were treated with DHA (positive control), Wilac D001, *E. coli*, or LPS under acute exposure conditions. GLP-1 secretion (ng/mL per mg protein) was quantified using ELISA. Data are expressed as mean ± SEM (n = 3). ** *p* < 0.01, **** *p* < 0.0001 vs. untreated control

**Fig. 6:**
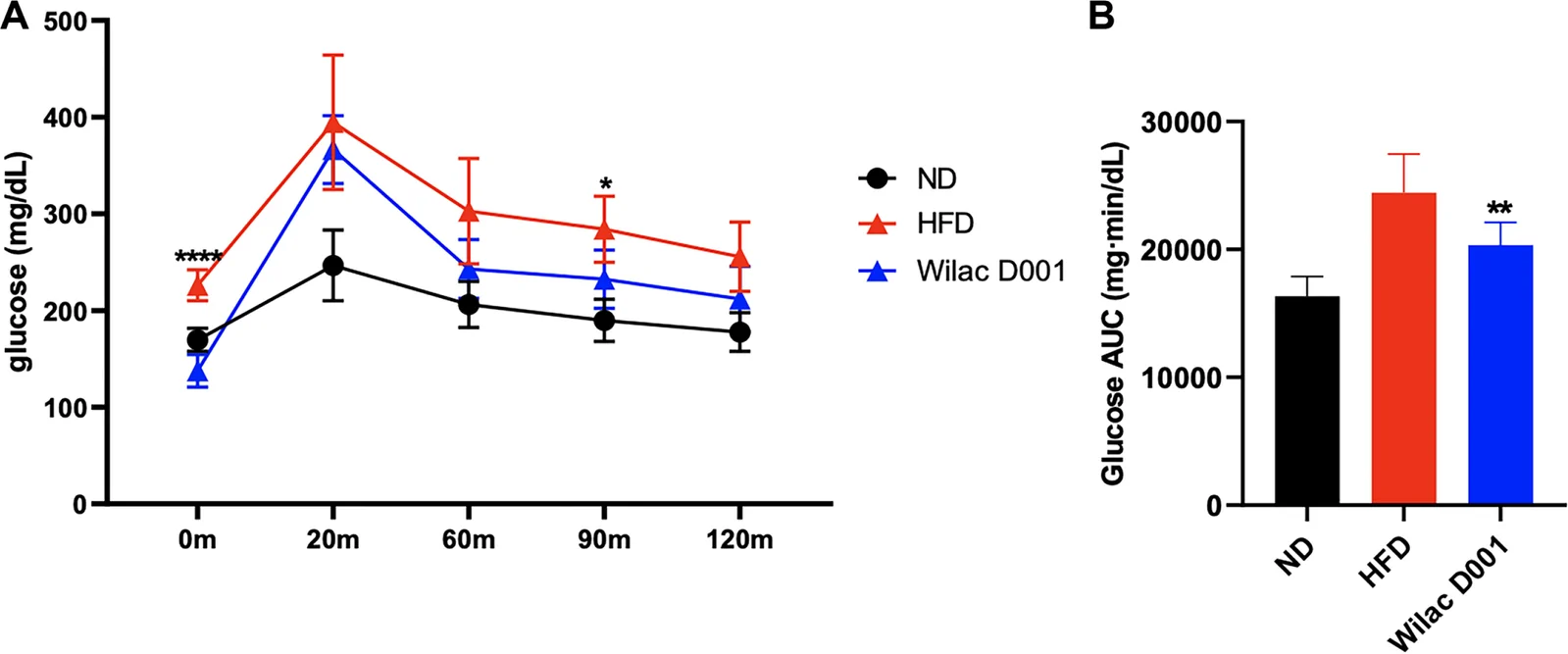
Effects of Wilac D001 on glucose tolerance in HFD-fed mice. (**A**) Oral glucose tolerance test (OGTT). Blood glucose levels were measured after an 18-h fast (0 min) and at 20, 60, 90, and 120 min following oral glucose administration. Data are presented as mean ± SD. Glucose time-course data were analyzed using two-way repeated-measures analysis of variance (ANOVA), followed by Sidak’s multiple-comparisons test. *p < 0.05 and ****p < 0.0001 versus the HFD group at the indicated time points. (**B**) Total glucose area under the curve (AUC) during the OGTT for each group. Data are presented as mean ± SD. Differences in AUC among groups were analyzed using one-way ANOVA followed by Tukey’s multiple-comparisons test. **p < 0.01 versus the HFD group. The glucose AUC was calculated using the trapezoidal rule and is expressed as mg·min/dL.

After 12 weeks of supplementation, significant reductions in body circumference were observed in the Wilac D001 group compared with the placebo group (Table 5 and Fig. 4). Waist circumference decreased by 4.38 ± 4.10 cm in the Wilac D001 group, and an increase of 1.37 ± 1.61 cm was observed in the placebo group (*p* < 0.0001). Hip circumference also decreased by 1.51 ± 1.65 cm in the Wilac D001 group but increased by 0.80 ± 1.24 cm in the placebo group (*p* < 0.0001).

The Wilac D001 group also showed significant reductions in upper arm and thigh circumference. Left and right arm circumference decreased by 1.39 ± 1.30 and 1.17 ± 1.03 cm, respectively, in the Wilac D001 group, whereas increases of 0.29 ± 1.34 and 0.27 ± 1.22 cm, respectively, were observed in the placebo group (both *p* < 0.0001). Similarly, left and right thigh circumference decreased by 1.27 ± 1.65 and 1.27 ± 1.32 cm, respectively, in the Wilac D001 group, whereas increases of 0.64 ± 0.86 cm and 0.52 ± 0.72 cm, respectively, were noted in the placebo group (both *p* < 0.0001).

### Changes in Energy Intake and Physical Activity

No significant between-group differences were observed in changes in total energy intake or physical activity during the 12-week intervention. At Week 12, total energy intake changed by -88.03 ± 599.12 kcal/day in the Wilac D001 group and 46.51 ± 608.37 kcal/day in the placebo group (*p* = 0.2455). Similarly, physical activity assessed using the GPAQ changed by 277.14 ± 2,223.20 and 223.33 ± 1,162.84 min/week in the Wilac D001 and placebo groups, respectively (*p* = 0.9780).

### Safety Outcomes

No serious adverse events or clinically significant adverse events were reported during the 12-week intervention period. Clinical laboratory evaluations, including hematological analysis, blood biochemistry, and urinalysis, were performed at baseline and at week 12, and all measured parameters remained within normal reference ranges throughout the study period. Among the hematological parameters, the eosinophil percentage significantly differed between the groups (*p* = 0.0490). The Wilac D001 group exhibited a mean decrease (-0.33% ± 1.38%), whereas the placebo group exhibited a mean increase (+0.11% ± 1.41%). However, the observed values in both groups remained within the normal clinical range, and these changes were not considered clinically meaningful. No statistically significant between-group differences were observed for the remaining laboratory parameters. Furthermore, no clinically significant abnormalities were identified in the vital signs or physical examination findings.

## Discussion

Probiotics are functional agents that may contribute to metabolic homeostasis by modulating the gut microbial environment, and increasing attention has been focused on their potential roles in obesity and metabolic health [14–16, 29]. In the present study, 12 weeks of Wilac D001 supplementation in overweight adults significantly reduced body weight, BMI, total body fat mass, abdominal fat, and anthropometric circumference measurements compared with placebo. Importantly, total body fat mass assessed by DEXA, the primary efficacy endpoint, was significantly reduced, whereas CT-based assessment showed significant decreases in total abdominal, visceral, and subcutaneous fat areas. In addition, the reductions in upper-arm and thigh circumferences were accompanied by regional reductions in fat mass, while no statistically significant between-group differences were observed in lean mass in either the arms or legs. These findings indicate that the observed reductions in limb circumference were accompanied by changes in regional adiposity and were not attributable solely to reductions in lean tissue. The consistency of these findings across anthropometric, DEXA, and CT-based assessments suggests that the effects of Wilac D001 were not limited to changes in body weight but were accompanied by reductions in both overall and regional adiposity. In particular, the reduction in visceral adiposity may be clinically relevant given the close relationship between excessive visceral fat accumulation, insulin resistance, and other obesity-related metabolic abnormalities [30].

Improvements in body composition were accompanied by changes in metabolic parameters. Triglyceride levels were significantly reduced compared with placebo, whereas total cholesterol, LDL cholesterol, and HbA1c were significantly reduced from baseline within the Wilac D001 group. These findings are generally consistent with previous clinical studies and meta-analyses reporting that probiotic supplementation may improve body weight, BMI, body fat, triglycerides, glycemic control, and indices related to insulin resistance [16, 25, 26]. However, because total cholesterol, LDL cholesterol, and HbA1c did not show significant between-group differences, these findings should be interpreted cautiously. Thus, the most consistent clinical effects of Wilac D001 were observed for body fat, abdominal adiposity, and triglycerides, whereas its effects on other metabolic parameters require further confirmation. Because probiotic effects may be strain-specific, findings from other strains cannot be directly extrapolated to Wilac D001 [14–16].

The clinical findings were supported by complementary preclinical observations. Wilac D001 significantly increased GLP-1 secretion in STC-1 cells, consistent with previous reports that certain probiotic strains can stimulate enteroendocrine GLP-1 secretion [9, 10, 18]. In the present HFD-induced mouse model of impaired glucose tolerance, Wilac D001 also improved OGTT responses and significantly reduced glucose AUC. Furthermore, previous animal studies investigating *Weissella confusa* WIKIM51 (Wilac D001) reported reductions in circulating glucose and insulin levels, improvements in glucose tolerance and insulin resistance, suppression of body weight gain and fat accumulation, and reductions in hepatic triglycerides [7, 8]. These studies also demonstrated activation of AMPK, increased expression of CPT1, PGC-1α, and PPARα, and suppression of FAS [7, 8], suggesting that regulation of fatty acid oxidation and lipogenesis may contribute to the metabolic effects of Wilac D001.

Nevertheless, the *in vitro* GLP-1 findings should be interpreted cautiously in relation to the clinical outcomes. STC-1 cells are a murine enteroendocrine tumor-derived cell line and therefore only partially reproduce the physiological characteristics and secretory responses of native human intestinal L-cells [17]. Moreover, the 15-min acute exposure used in the cell experiment differs substantially from the 12-week supplementation period in the clinical trial. Thus, increased GLP-1 secretion in STC-1 cells should not be interpreted as direct evidence that Wilac D001 increases circulating GLP-1 in humans or that GLP-1 directly mediates the observed reductions in adiposity and triglycerides.

Notably, no significant between-group differences in changes in total energy intake or physical activity were observed during the clinical intervention. Therefore, the reductions in body fat and triglycerides cannot be readily explained by differential changes in measured energy intake or physical activity between the groups and are unlikely to result solely from GLP-1-mediated appetite suppression. Probiotics may influence host metabolism through multiple pathways within the gut–metabolic axis, including microbial metabolites such as short-chain fatty acids, bile acid signaling, intestinal barrier function, inflammatory responses, intestinal lipid digestion, absorption and excretion, and hepatic lipid synthesis and fatty acid oxidation [14, 20, 22]. These mechanisms may act in concert, and the previously reported activation of AMPK and fatty acid oxidation-related pathways by *W. confusa* WIKIM51 (Wilac D001) [7, 8] provides preclinical support for mechanisms beyond appetite regulation.

This study has limitations regarding the direct mechanistic interpretation of the clinical findings. Although circulating GLP-1, insulin sensitivity, and other mechanistic biomarkers were not directly assessed in the clinical trial, previous animal studies of *W. confusa* WIKIM51 (Wilac D001) demonstrated reductions in circulating glucose and insulin levels, improvements in insulin resistance, reductions in triglyceride and cholesterol levels, and modulation of lipid metabolism-related pathways [7, 8]. These preclinical findings provide biological and mechanistic support for the improvements in body fat and metabolic parameters observed in the present clinical trial. Nevertheless, direct mechanistic studies in humans, including measurements of circulating GLP-1, insulin sensitivity, gut microbiota composition, and related metabolic biomarkers, are needed to determine whether these pathways contribute to the clinical effects of Wilac D001.

Collectively, 12 weeks of Wilac D001 supplementation significantly reduced body weight, total body fat mass, abdominal adiposity, and triglyceride levels in overweight adults. The STC-1 and animal findings, together with previous animal studies of *W. confusa* WIKIM51 (Wilac D001) demonstrating improvements in glucose, insulin, and lipid metabolism [7, 8], provide complementary biological and mechanistic support for the metabolic improvements observed in the clinical trial. These findings suggest that Wilac D001 may be a promising functional probiotic candidate for reducing body fat accumulation and improving metabolic health, although further independent clinical studies are warranted to confirm these effects and clarify the underlying mechanisms.

## Conclusion

Wilac D001 increased GLP-1 secretion in STC-1 cells and improved glucose tolerance in high-fat diet-fed mice, providing complementary biological support for its potential metabolic effects. In the clinical trial, 12 weeks of Wilac D001 supplementation significantly reduced body weight, total body fat mass, abdominal adiposity, and triglyceride levels compared with placebo in overweight adults. These findings suggest that Wilac D001 may be a promising functional probiotic candidate for reducing body fat accumulation and improving metabolic health. Further independent clinical studies are warranted to confirm these effects and clarify the underlying mechanisms.

## Figures and Tables

**Table 1:** Participants’ characteristics.

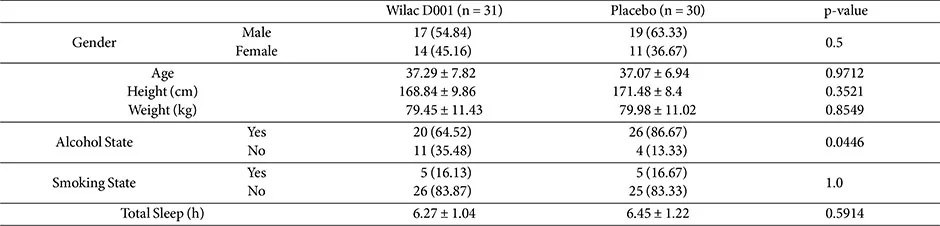

**Table 2:** Changes in body composition parameters after 12 weeks of intervention with Wilac D001 or placebo.

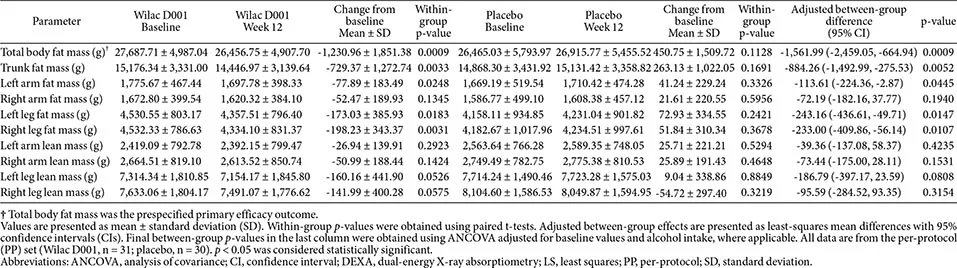

**Table 3:** Changes in serum lipid parameters (total cholesterol, triglycerides, and HbA1c).

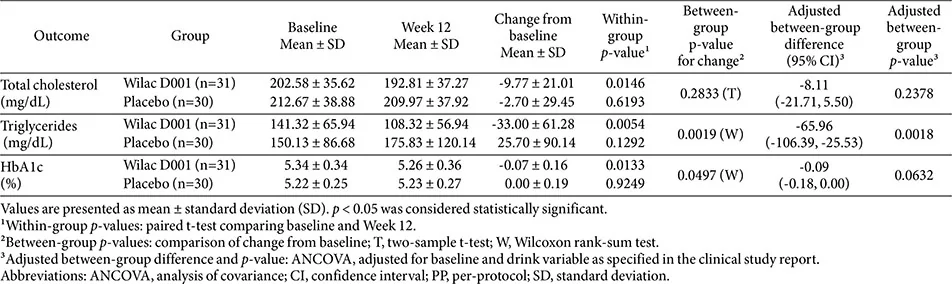

**Table 4:** Changes in total, visceral, and subcutaneous abdominal fat area (cm^2^) assessed by CT after 12 weeks.

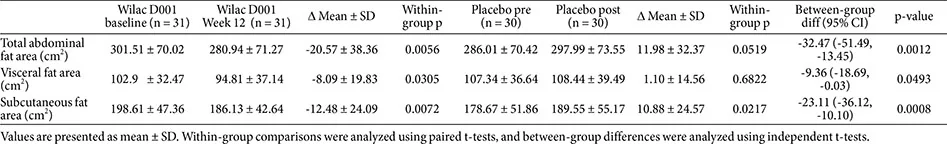

**Table 5:** Changes in waist, hip, upper arm, and thigh circumferences.